# MicroRNA-212 suppresses the proliferation and migration of osteosarcoma cells by targeting forkhead box protein A1

**DOI:** 10.3892/etm.2021.10409

**Published:** 2021-07-09

**Authors:** Jian Liu, Bohua Chen, Bin Yue, Junde Yang

Exp Ther Med 12:4135–4141, 2016; DOI: 10.3892/etm.2016.3880

Following the publication of this paper, it was drawn to the Editors' attention by a concerned reader that the Transwell cell migration data shown in Figs. 4 and 9 were strikingly similar to data appearing in different form in other articles by different authors. Owing to the fact that the contentious data in the above article had already been published elsewhere, or were already under consideration for publication, prior to its submission to *Experimental and Therapeutic Medicine*, the Editor has decided that this paper should be retracted from the Journal. The authors were asked for an explanation to account for these concerns, but the Editorial Office did not receive any reply. The Editor apologizes to the readership for any inconvenience caused.

